# The relationships between cognitive function, literacy and HIV status knowledge among older adults in rural South Africa

**DOI:** 10.1002/jia2.25457

**Published:** 2020-03-23

**Authors:** Molly Rosenberg, F. Xavier Gómez‐Olivé, Ryan G. Wagner, Julia Rohr, Collin F. Payne, Lisa Berkman, Kathleen Kahn, Stephen Tollman, Till Bärnighausen, Lindsay C. Kobayashi

**Affiliations:** ^1^ Department of Epidemiology and Biostatistics Indiana University School of Public Health‐Bloomington Bloomington IN USA; ^2^ MRC/Wits Rural Public Health and Health Transitions Research Unit (Agincourt) School of Public Health Faculty of Health Sciences University of the Witwatersrand Johannesburg South Africa; ^3^ INDEPTH Network Accra Ghana; ^4^ Umeå Centre for Global Health Research Division of Epidemiology and Global Health Department of Public Health and Clinical Medicine Umeå University Umeå Sweden; ^5^ Center for Population and Development Studies Harvard University Cambridge MA USA; ^6^ School of Demography, Research School of Social Sciences Australian National University Canberra Australia; ^7^ Africa Health Research Institute Durban South Africa; ^8^ Heidelberg Institute of Global Health Faculty of Medicine and University Hospital University of Heidelberg Heidelberg Germany; ^9^ Department of Epidemiology School of Public Health University of Michigan Ann Arbor MI USA

**Keywords:** HIV testing, older adults, South Africa, cognitive function, literacy, education

## Abstract

**Introduction:**

Although HIV prevalence is exceptionally high in South Africa, HIV testing rates remain below targeted guidelines. Older adults living with HIV are substantially more likely to remain undiagnosed than younger people. Cognitive function and literacy could play key roles in HIV status knowledge due to the decision‐making processes required around weighing the costs and benefits of testing, navigating testing logistics and processing results. We aimed to assess the independent relationships among each of cognitive function, literacy and education with HIV status knowledge in a population‐based sample of older adults living in a rural South African community with high HIV prevalence.

**Methods:**

We analyzed data from a population‐based study of 5059 men and women aged 40 years and older in rural South Africa (Health and Aging in Africa: A Longitudinal Study of an INDEPTH community (HAALSI)). HAALSI surveys, conducted between 2014 and 2015, queried self‐reported literacy, educational attainment and HIV status knowledge. Laboratory tests were conducted to assess true HIV sero‐status. Cognitive function was assessed with a battery of cognitive tests measuring time orientation, immediate and delayed recall, and numeracy and coded using confirmatory factor analysis as a z‐standardized latent variable. We estimated the relationship between the outcome of HIV status knowledge and each of three exposures: (1) latent cognitive z‐score, (2) literacy and (3) education, using confounder‐adjusted modified Poisson regression models in the study population overall and stratified by HIV sero‐status.

**Results:**

We found that HIV status knowledge was higher among those with higher cognitive z‐scores (adjusted Prevalence Ratio (aPR) (95% CI): 1.18 (1.14, 1.21) per standard deviation unit), and among literate participants (aPR (95% CI): 1.24 (1.16, 1.32) vs. non‐literate participants). Taken together, the associations with literacy and cognitive function completely attenuated the otherwise positive association between educational attainment and HIV status knowledge. The magnitudes of effect were generally similar among laboratory‐confirmed HIV‐negative and HIV‐positive participants.

**Conclusions:**

Campaigns that target older adults in rural South Africa with HIV testing messages should carefully consider the cognitive and literacy levels of the intended audience. Innovations to ease the cognitive load associated with HIV testing could prove fruitful to increase HIV status knowledge.

## 1 Introduction

HIV testing and status knowledge are critical first steps in identifying people living with HIV and transitioning them into treatment and care. South Africa is one of the countries hardest hit by the HIV epidemic where approximately seven million people are living with HIV, and regional prevalence estimates range from 18% to 40% 1, 2. Older adults in particular make up an increasing proportion of people living with HIV because of the success of large‐scale HIV treatment at prolonging life, and because of ongoing sexual risk 3, 4, 5, 6, 7, 8, 9. The HIV prevalence for South Africans over age 50 is approximately 9% 3, 6, 10, with even higher prevalence estimates observed in rural settings (e.g. 17% 11 and 20% 9). Although HIV prevalence is exceptionally high in South Africa, HIV testing rates remain below targeted guidelines 12. Older South Africans living with HIV are substantially more likely to remain undiagnosed than younger people. Of all South Africans age 60 and older living with HIV in 2012, approximately 40% had never been tested for HIV 12, 13.

Older adults in South Africa had limited access to educational opportunities as children and young adults, and, as such, often have low educational attainment. During the legislated racial segregation under Apartheid (1948 to 1994), Black South Africans were provided with extremely limited public services and had poor access to education 14, 15. Over 25% of South Africans age 60 and older have had no formal education; the proportion reaches close to 40% among Black South Africans 16. It is well‐established that higher educational attainment is an important predictor of HIV testing and HIV status knowledge in sub‐Saharan Africa 17, 18, 19, 20, 21. Understanding the mechanisms through which education may influence status knowledge could provide insight into the low testing rates in older adults and identify potential interventions for increasing HIV status knowledge in this vulnerable population.

One of the mechanisms through which education could influence HIV testing and status knowledge is through its effects on cognitive function 22. Cognition describes the set of mental processes related to knowledge and understanding. Cognitive function is essential for communication through speech and language; in making decisions through the integration of new and existing knowledge, reasoning and executive functioning; and underpins the decisions that lead to behaviours 23. Cognitive function generally increases with exposure to formal education 24, but declines with age 25, 26, 27. The decision to undergo HIV testing and the subsequent processing of status knowledge likely requires a relatively high cognitive load to understand the meaning of testing, accurately perceive risk, weigh the costs and benefits and navigate testing logistics 28. The relationship between cognitive function and other areas of engagement in medical care – treatment choice 29 and treatment adherence 30, 31, 32, 33 – has been established. However, we are not aware of previous studies that have examined the relationship between cognitive function and HIV status knowledge.

**Figure 1:**
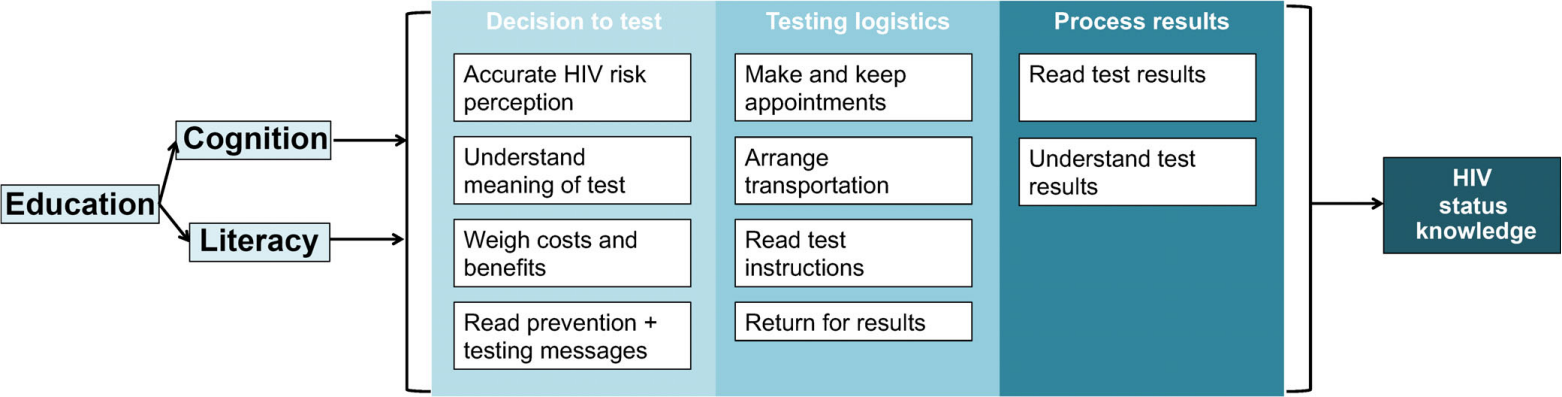
Conceptual framework of the relationship between education, cognition, literacy and HIV status knowledge.

Literacy, the ability to read and write, could also influence HIV status knowledge independently of or in combination with cognitive function (see Figure 1). In contrast to cognitive function, literacy remains relatively stable with age after being established early in life through exposure to formal education. In South Africa, of those with no formal education, only 16% are able to read without difficulty 16. Like cognition, there are likely to be key literacy requirements to comprehend and process HIV testing messages, testing instructions and results 34, 35. Also like cognition, the relationships between literacy and some areas of engagement in medical care have been established 36, 37, 38, 39, but the few studies that have examined the relationship between literacy and HIV testing have produced mixed results 40, 41, 42.

In this study, we assessed the relationship among education, cognition, literacy and HIV status knowledge in a sample of older adults living in a community with high HIV prevalence. Using data from the baseline survey of a population‐based cohort in rural South Africa, we: (i) measured the associations among cognition, literacy and HIV status knowledge; (ii) determined how much of the relationship between education and knowledge of HIV status is accounted for by cognitive function and literacy; and (iii) quantified heterogeneity of these relationships by true HIV status.

## 2 Methods

### 2.1 Study population

We analyzed data from the 2014 to 2015 baseline assessments of “Health and Aging in Africa: A Longitudinal Study of an INDEPTH community” (HAALSI) 43. HAALSI is a population‐based study of men and women aged 40 years and older in the rural Agincourt sub‐district of Mpumalanga province, South Africa. The study was designed to broadly characterize the physical and cognitive functioning of an aging rural South African population. The HAALSI study is nested in the Agincourt Health and socio‐Demographic Surveillance System (HDSS) of the Medical Research Council/ Wits University Rural Public Health and Health Transitions Research Unit 44, 45. In 2014 to 2015, 5059 men and women age 40 years and older enrolled in the study and provided extensive survey and laboratory data on (i) Physical and cognitive functioning, (ii) Cardiometabolic health, (iii) Economic well‐being and (iv) HIV and HIV risk.

Participants were randomly selected from within the 2013 Agincourt HDSS sampling frame, based on age and permanent resident status. A total of 12,875 people met the age and permanent resident eligibility criteria. Of them, 6281 were randomly selected for potential participation in the HAALSI cohort. Of the 5890 who remained eligible by the time of data collection (did not die or out‐migrate in the interim), 5059 enrolled in the study, yielding an 85.9% response rate 43. Study staff approached potential participants at their home and provided them with verbal and written information about the study. Study staff then asked participants to provide signed informed consent in xiTsonga, the local language, or in English. A witness was requested for the consent process with illiterate participants and they signed using a fingerprint. Ethical approval for HAALSI was obtained from the University of the Witwatersrand Human Research Ethics Committee (#M141159), the Harvard T.H. Chan School of Public Health Office of Human Research Administration (#13‐1608), and the Mpumalanga Provincial Research and Ethics Committee.

### 2.2 Key measures

All variables were collected in HAALSI baseline surveys, physical and cognitive function assessments and biological testing. Survey data were collected by trained local fieldworkers using a Computer Assisted Personal Interviews platform in the local xiTsonga language. We assessed three exposures:

1. Cognitive function: a continuous z‐standardized latent variable (mean 0, standard deviation (SD) 1) representing time orientation (ability to state the correct day, month, year and South African president), immediate and delayed recall of 10 words read out loud by the interviewer, and numeracy (ability to count from 1 to 20 and to finish a number skip pattern) 46. The cognitive function z‐score was derived using confirmatory factor analysis (CFA) with a robust weighted least squares estimator, which used the common covariation between the individual cognitive test items to generate a latent cognitive function z‐score. This method reduces measurement error, weights the individual cognitive test items according to their contribution to underlying cognitive function (unlike a simple summary score), and allows for non‐linear relationships between cognitive test items and underlying cognitive function 47, 48 Our CFA model is described in more detail elsewhere 37.
2. Literacy: self‐reported ability to read or write (yes/no)
3. Education: self‐reported highest year of education completed, coded as “no formal education” vs. “some formal education” as there was a threshold relationship with the likelihood of HIV testing at this point. We also conducted sensitivity analyses with education coded continuously as years of education, and according to degree level (no education; some primary (one to seven years); some secondary (eight to eleven years); and secondary or higher (12+ years)).

Our outcome of interest was self‐reported knowledge of HIV status, dichotomized into categories of individuals who tested and knew their status versus individuals who had either not tested or had tested but did not know their status. Among those who reported HIV testing, a follow‐up question about timing of most recent test was asked. We considered participants with an HIV test in the previous six months as recent testers.

We also considered several key socio‐demographic variables as potential confounders: sex (male versus female), age (40 to 49, 50 to 59, 60 to 69, 70 to 79, 80+), country of birth (South Africa, Mozambique or other), marital status (never married, separated/divorced, widowed, currently married or cohabitating) and household asset index (weighted index based on ownership of consumer durables, livestock and housing construction, categorized in quintiles). We considered true HIV status as a potential effect measure modifier. Laboratory‐confirmed HIV status was determined through screening and confirmatory HIV enzyme‐linked immunosorbent assays conducted using standard laboratory practices on prepared dried blood spots 49.

### 2.3 Statistical analysis

We summarized sociodemographic characteristics of the sample overall, and by HIV status knowledge. Poisson regression models with robust standard errors and a log link for binary outcome data were used to estimate the associations between knowledge of HIV status and each of cognitive z‐score, literacy, and education as the independent variables in a series of three models: model set 1 included the three independent variables in separate, unadjusted models; model set 2 additionally included the *a priori* selected covariates in the separate models; and, model set 3 mutually adjusted for all three independent variables in the same model, along with covariates 50. We repeated this modeling strategy with the outcome restricted to knowledge of HIV status from a recent HIV test (within the last six months). For this secondary analysis, we removed those who self‐reported HIV+ status from a test more than six months old. We calculated and visualized the age‐ and sex‐standardized mean cognitive function z‐scores according to combined self‐reported and true HIV status, based on laboratory‐confirmed results. We further incorporated a multiplicative interaction term between each of the three independent variables and laboratory‐confirmed HIV status to assess whether true HIV status modified any of the observed relationships. We assessed potential multicollinearity of the three exposure variables by examining their variance inflation factors (VIFs).

## 3 Results

Of the 5059 participants enrolled in the HAALSI study from 2014 to 2015, 54% were women, 51% were currently married or cohabitating, and 70% were born in South Africa (nearly all of the remaining 30% were born in neighboring Mozambique 44) (Table 1). Median age was 61 years (IQR: 52 to 71 years). Nearly half of the population (46%) received no formal education, and 42% were illiterate. Dried blood spot (DBS) laboratory testing conducted in the course of the study confirmed an HIV prevalence of 23.0% (95% CI: 21.8, 24.2) among the participants.

**Table 1:** Sample characteristics, overall and according to HIV status knowledge, HAALSI, South Africa, 2015

Characteristic	Overall 5059 (100%)	Knows HIV status^a^ 2847 (57%)	Does not know HIV status^a^ 2134 (43%)	*p*‐value
Sex (n = 5059)				
Male	2345 (46%)	1282 (45%)	1026 (48%)	0.03
Female	2714 (54%)	1565 (55%)	1108 (52%)	
Age group (n = 5059)				
40 to 49	918 (18%)	608 (21%)	299 (14%)	<0.0001
50 to 59	1410 (28%)	919 (32%)	469 (22%)	
60 to 69	1304 (26%)	737 (26%)	542 (25%)	
70 to 79	878 (17%)	398 (14%)	470 (22%)	
80+	549 (11%)	185 (7%)	354 (17%)	
Marital status (n = 5055)				
Never married	290 (6%)	150 (5%)	135 (6%)	<0.0001
Separated/divorced	650 (13%)	400 (14%)	242 (11%)	
Widowed	1540 (30%)	780 (27%)	732 (34%)	
Currently married	2575 (51%)	1515 (53%)	1023 (48%)	
Country of birth (n = 5054)				
South Africa	3528 (70%)	2026 (71%)	1450 (68%)	0.01
Mozambique or other	1526 (30%)	818 (29%)	682 (32%)	
Wealth asset quintile (n = 5059)				
1 (poorest)	1406 (21%)	530 (19%)	449 (23%)	<0.0001
2	1001 (20%)	547 (19%)	439 (21%)	
3	991 (20%)	571 (20%)	406 (19%)	
4	1007 (20%)	581 (20%)	411 (19%)	
5 (wealthiest)	1014 (20%)	618 (22%)	379 (18%)	
Education (n = 5042)				
None	2307 (46%)	1098 (39%)	1164 (55%)	<0.0001
At least one year	2735 (54%)	1740 (61%)	962 (45%)	
Literate (n = 5046)				
No	2108 (42%)	967 (34%)	1099 (52%)	<0.0001
Yes	2948 (58%)	1879 (66%)	1033 (48%)	
Latent cognitive function score (n = 4927)				
Mean (SD)	0.00 (1.00)	0.20 (0.02)	−0.27 (0.02)	<0.0001
Dried blood spot HIV result (n = 4560)				
Negative	3512 (77%)	1866 (70%)	1638 (87%)	<0.0001
Positive	1048 (23%)	799 (30%)	248 (13%)	

a The total denominator for HIV status knowledge variable was n = 4981. Twenty participants did not respond to the question on whether they had ever tested for HIV (n = 10 refused and n = 10 did not know). A further 58 participants were removed because they reported the inconsistent responses that they knew their HIV status but had never previously tested for HIV.

Just over half (57%) of the participants reported previous HIV testing with knowledge of their HIV status. Of those who reported status knowledge, only 12% self‐reported their status as HIV+. Of those with laboratory‐confirmed HIV infections, 76% reported HIV status knowledge, but just over half (52%) self‐reported their status as HIV+. Those with HIV status knowledge were more likely to have laboratory confirmed HIV infections than those without HIV status knowledge. Participants with HIV status knowledge also tended to be younger, and to be categorized in wealthier household asset quintiles. They were slightly more likely to be women (55% vs 52%) and to be of South African descent (71% vs. 68%). Participants without status knowledge were more likely to be widowed (34% vs. 27%).

Participants with formal education, participants who were literate and participants with higher cognitive z‐scores were more likely to have HIV status knowledge (Table 2). In univariate analysis, participants who received formal education were 30% more likely to report HIV status knowledge (Prevalence Ratio (PR) (95% CI): 1.30 (1.23, 1.37)); participants who were literate were 35% more likely to report HIV status knowledge (PR (95% CI): 1.35 (1.28, 1.43)); and for every SD increase in latent cognitive z‐score there was a corresponding 22% increase in likelihood of HIV status knowledge (PR (95% CI): 1.22 (1.19, 1.25)).

**Table 2:** Adjusted prevalence ratios (PRs) and 95% confidence intervals (CIs) for HIV status knowledge outcome, HAALSI, South Africa, 2015

Exposure variables	Formal education (At least one year vs. none)	Literate (Yes vs. No)	Latent cognitive z‐score (Per SD increase)
	PR (95% CI)	PR (95% CI)	PR (95% CI)
Full sample (n = 4446)			
Unadjusted	1.30 (1.23, 1.37)	1.35 (1.28, 1.43)	1.22 (1.19, 1.25)
Covariate adjusted^a^	1.18 (1.11, 1.26)	1.24 (1.16, 1.32)	1.18 (1.14, 1.21)
Fully adjusted^b^	1.04 (0.96, 1.11)	1.08 (1.00, 1.16)	1.15 (1.12, 1.18)
HIV negative (n = 3415)^c^			
Fully adjusted^b^	1.04 (0.95, 1.14)	1.06 (0.96, 1.17)	1.19 (1.14, 1.23)
HIV positive (n = 1031)^c^			
Fully adjusted^b^	1.06 (0.96, 1.18)	1.11 (1.00, 1.24)	1.05 (1.01, 1.10)

a Covariates are age, sex, marital status, country of birth and household wealth asset quintile

b adjusted for covariates with mutual adjustment for education, literacy and latent cognitive z‐score

c multiplicative statistical interaction terms between DBS HIV result and each of education, literacy and latent cognitive function score had *p*‐values of: 0.10 (education), 0.13 (literacy) and <0.0001 (cognitive z‐score).

For the education, literacy and cognitive function exposures, findings were attenuated modestly but remained statistically significant in model set 2 after adjustment for key socio‐demographic variables (age, sex, marital status, country of birth and wealth asset quintile). Results were attenuated again after mutual adjustment for education, literacy and latent cognitive score with the socio‐demographic controls in model set 3. After mutual adjustment, literacy and cognitive scores both remained significantly and positively associated with HIV status knowledge (aPR_literacy_ (95% CI): 1.08 (1.00, 1.16); aPR_cognition_ (95% CI): 1.15 (1.12, 1.18)). The association between education and HIV status knowledge moved much closer to the null with confidence intervals spanning the null after adjustment for cognition and literacy (aPR_education_ (95% CI): 1.04 (0.96, 1.11)). VIF values were 1.99 for education, 2.20 for literacy, 1.56 for cognitive function (Mean VIF = 1.92), indicating low multicollinearity among variables. The sensitivity analyses with education coded continuously and according to degree level showed similar results (data not shown). We found similar trends, though stronger in magnitude, when we restricted the outcome to HIV status knowledge based on a recent HIV test in the last six months (Table 3).

**Table 3:** Adjusted prevalence ratios (PRs) and 95% confidence intervals (CIs) for HIV status knowledge outcome based on an HIV test within the past six months,^a^ HAALSI, South Africa, 2015

Exposure variables	Formal education (At least one year vs. none)	Literate (Yes vs. No)	Latent cognitive z‐score (Per SD increase)
	PR 95% CI	PR 95% CI	PR 95% CI
Full sample (n = 4113)			
Unadjusted	1.54 (1.32, 1.80)	1.75 (1.49, 2.05)	1.37 (1.29, 1.47)
Covariate adjusted^b^	1.22 (1.01, 1.48)	1.50 (1.23, 1.82)	1.25 (1.16, 1.36)
Fully adjusted^c^	0.91 (0.73, 1.13)	1.36 (1.07, 1.72)	1.20 (1.10, 1.31)

a After excluding those who refused or did not know their testing history, those who refused or did not know the date of their most recent test, those who reported being HIV positive based a test >6 months ago, and those with missing covariate data, the total denominator for this analysis was n = 4113

b covariates are age, sex, marital status, country of birth, and household wealth and asset quintile

c adjusted for covariates with mutual adjustment for education, literacy and latent cognitive z‐score.

In the models stratified by HIV status, results in the HIV‐positive and HIV‐negative participants were fairly similar to each other and to the results in the overall sample (Table 2). Regardless of sero‐status, in the fully adjusted models, the association between education and HIV status knowledge was fully attenuated by literacy and cognitive function. The *p*‐values associated with the multiplicative interaction term between DBS HIV result and each of education and literacy were 0.10 and 0.13 respectively. However, a minor difference by HIV status was noted for cognitive function. The positive relationship between cognitive function and testing uptake was slightly stronger among the HIV‐ negative participants (aPR (95% CI): 1.19 (1.14, 1.23)) compared to the HIV‐positive participants (aPR (95% CI): 1.05 (1.01, 1.10)). The *p*‐value associated with the multiplicative interaction term between DBS HIV result and cognitive score was <0.0001.

**Figure 2:**
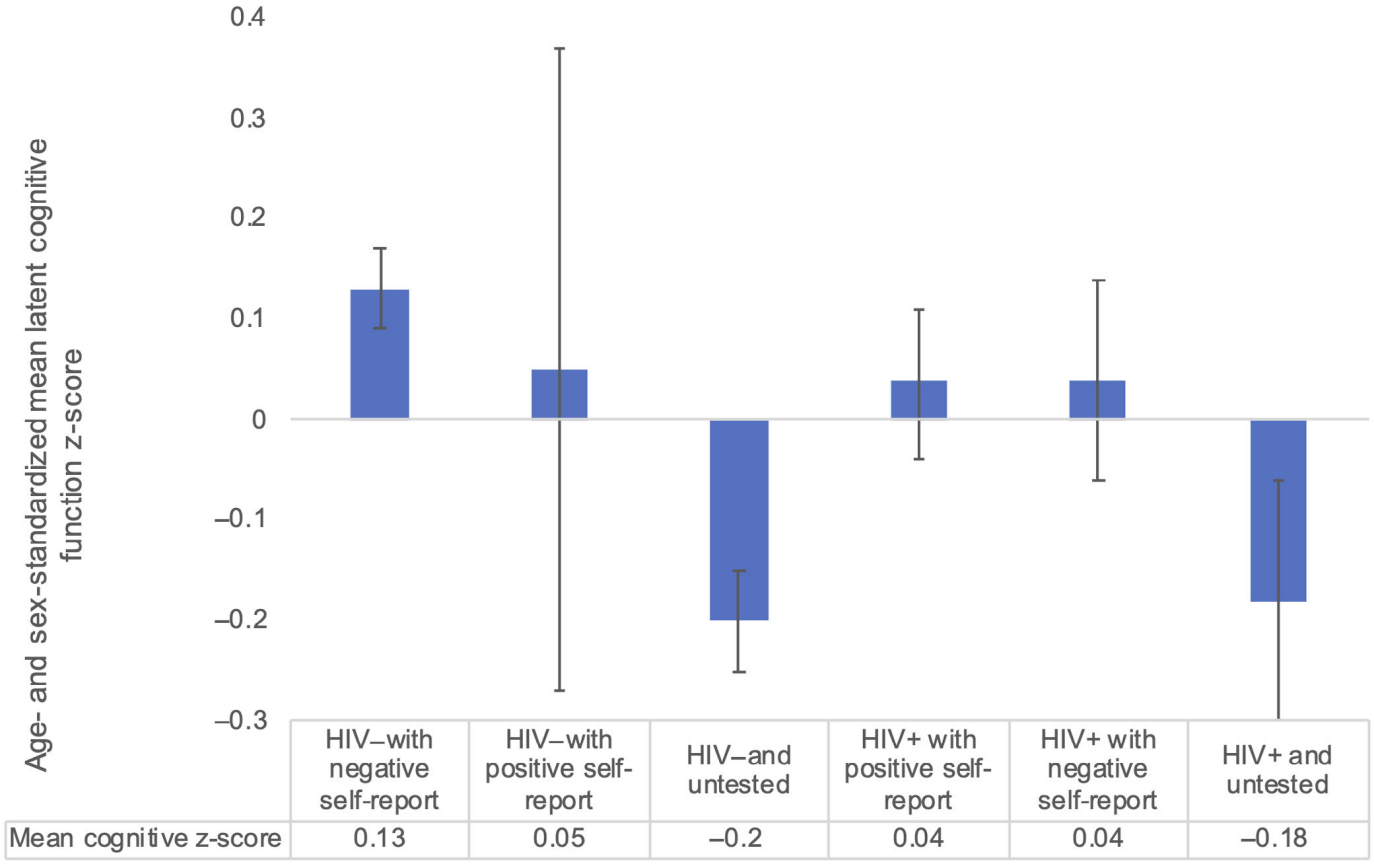
Age‐ and sex‐standardized mean latent cognitive scores by HIV testing status (combination of self‐reported and dried blood test result). N for each category: 2068 (0.13), 29 (0.05), 1400 (−0.20), 535 (0.04), 307 (0.04), 201 (−0.18).

In general, cognitive scores tended to be more predictive of HIV testing history (yes/no) than of concordance between self‐reported and actual HIV status (Figure 2). After age‐ and sex‐standardization, the lowest mean cognitive scores were among those who were HIV negative but untested (−0.20) and those who were HIV positive but untested (−0.18). Those who had previously tested for HIV tended to have higher cognitive scores regardless of their true sero‐status, or whether they accurately self‐reported their status. Similar cognitive scores were observed for participants who were HIV negative and self‐reported correct HIV‐negative status knowledge (0.13) or incorrect HIV‐positive status knowledge (0.05), and for participants who were HIV positive and self‐reported correct HIV‐positive status knowledge (0.04) or incorrect HIV‐ negative status knowledge (0.04). In sum, cognitive scores were associated with actual HIV testing behaviours more strongly than whether or not the participants had correct knowledge about their HIV status.

## 4 Discussion

In this study of older adults in rural South Africa from 2014 to 2015, we found that HIV status knowledge was higher among those with higher cognitive scores, those who reported being literate, and those with any level of formal education. Considering all three variables together, the association between education and HIV status knowledge was explained by higher levels of literacy and cognitive function, suggesting that cognitive function and literacy are the main mechanisms through which education may benefit HIV testing uptake and HIV status knowledge. We also observed stronger associations when we restricted the HIV status knowledge outcome to those based on recent HIV tests. This finding underscores the potential importance of this education‐cognitive function‐literacy pathway to HIV status knowledge based on best testing practices.

A growing body of evidence has found low uptake of HIV testing in older adults overall 12, 51. In South Africa, those who do test tend to test late, motivated by symptomatic infections or a positive partner 52. Many of those who do not test cite a lack of perception of being “at‐risk” as a reason for not testing 51. The cognitive and literacy factors we identified in this study may be important underlying explanations for the low testing uptake in older South Africans. Future studies should seek to better understand how cognitive function and literacy could influence accurate HIV risk perception, and the behaviours that underlie HIV risk 53.

We found little evidence for difference in the relationship between cognitive function and HIV status knowledge by true HIV status. Although we hypothesized that poor cognitive function could reduce the likelihood of HIV testing, we anticipated the potential for a reverse causal relationship as well. A sequela of long‐term HIV infection is HIV‐associated neurocognitive disorder (HAND) 54. HAND manifests as cognitive impairment, ranging from mild to severe and is caused by viral involvement in the central nervous system. Although antiretroviral therapy (ART) coverage is high in this study population for those with HIV status knowledge (95%) 55, it is plausible that HIV‐positive adults, particularly those without status knowledge, may have lower cognitive scores directly resulting from long‐term, uncontrolled infection. Due to this causal relationship, we anticipated that cognitive function would be particularly low in HIV‐positive participants who were unaware of their status and, therefore, unlikely to have therapy‐controlled infections. Thus, we anticipated that the relationship between cognitive function and HIV testing uptake would be stronger among HIV‐positive compared to HIV‐positive participants. However, the opposite relationship was observed: the relationship between cognitive function and HIV testing was slightly stronger in the HIV‐negative participants, though present for HIV‐positive participants as well. Survival bias is one potential explanation for these unexpected results, if the HIV‐positive adults who survive to older ages are cognitively healthier than the general population of HIV‐negative older adults. Future research should prioritize (1) longitudinal data collection to better establish the temporal relationships between cognitive function and HIV infection, and (2) analysis of ART uptake as a potentially informative covariate.

Several aspects of the study design and measures warrant a cautious interpretation of our findings. First, the cross‐sectional nature of the data leads to uncertainty around the temporal relationships between key variables. In particular, we have interpreted our findings under the assumption that education early in life causes improved cognitive function in later life. An alternative explanation is that the limited educational opportunities for Black South Africans during Apartheid were selectively given to those with stronger childhood cognitive abilities, and that these stronger cognitive abilities were maintained at older ages. Longitudinal cohort data across the life course would be valuable in untangling the temporal relationships between education and cognitive function, and their respective relationships with outcomes including HIV testing uptake and status knowledge.

Second, HIV testing and status knowledge were self‐reported and could be subject to social desirability bias. This would be problematic if the potential for social desirability bias differed by cognitive capacity, a plausible scenario in our study. Future studies should seek to use more objective measures of HIV testing history, perhaps through linkages to local health clinics and testing facilities. Our literacy measure was also self‐reported. Previous research in settings where illiteracy is rare, such as the United States, demonstrates that study participants in health research often under‐report illiteracy due to stigma or shame 56, 57. While illiteracy may also be under‐reported in our study population, it was a normative situation as nearly half of participants reported that they could not read or write. We believe that, in this community, illiteracy is unlikely to be associated with the same stigma that it is in contexts, such as the United States, where it is rare and non‐normative. Future waves of the HAALSI study have incorporated in‐depth literacy assessments, which will allow us to validate the self‐reported literacy measure and to use more fine‐grained measures of literacy skills. Future research should also consider the effects of domain‐specific literacy skills, such as health literacy.

Third, the global cognitive score we used to measure cognitive function may obscure any variation in relationship between HIV status knowledge and more specific cognitive sub‐domains. Our cognitive score captured time orientation, episodic memory and numeracy, but we did not have measures of other domains. More specific cognitive tests in domains such as executive function, risk perception, and risk proclivity could provide a more nuanced understanding of these important relationships 53.

Finally, our data on HIV status knowledge in older South Africans were collected in 2014 to 2015. It is possible that trends in testing behaviours have changed since the data were collected. However, more recently published studies continue to highlight the unacceptably high rates of undiagnosed HIV cases in South Africa 58, 59. Furthermore, the most recent South African Prevalence Survey in 2017 concluded that although some improvements in HIV testing rates were observed since 2012, interventions to change risk perceptions and improve treatment literacy were still critical to improve the efficacy of widespread HIV testing and linkage to care 60. The potential for issues with generalizability to current populations remains, however, and the findings from this study should be updated with more recent data as they become available.

## 5 Conclusions

Our findings add to the growing body of evidence that interventions tailored to the unique HIV prevention needs of older adults are critically needed to prevent the spread of infection in this understudied group. Contrary to stereotypes about sexual activity in older age, older adults maintain sexual relationships and engage in behaviours that are consistent with the onward transmission of HIV 9, 61, 62. Thus, interventions that lead to accurate HIV risk perception, regular HIV testing and correct HIV status knowledge in older adults are necessary. Our findings point to several potential intervention targets. At minimum, campaigns that target older adults with HIV testing messages should carefully consider the cognitive and literacy constraints of their audience, and shape their messaging accordingly. An evaluation of a recent campaign designed to increase South African HIV testing via mass‐media promotion and door‐to‐door distribution of pamphlets found that although testing uptake increased, the disparity in testing uptake by education actually widened during the campaign 20. Innovations to ease the cognitive load associated with HIV testing could facilitate uptake of HIV testing by older adults. Older adults with low or no formal education experience would also be likely to benefit from non‐formal opportunities to improve their literacy. From a life course perspective and assuming a causal relationship, our findings underscore the profound and long‐lasting impacts of education on later‐life health outcomes. The elevated cognitive and literacy capacity observed in those with formal education were associated with increased HIV testing behaviours in later adulthood. Investments in education with the goal of increasing the cognitive and literacy capacity of a population as it ages will continue to pay long‐term dividends in improved health.

## Competing interest

The authors declare that they have no conflicts of interest.

## Authors’ contributions

MR and LCK conceived the study, conducted the analysis and wrote the first draft of the manuscript, with input and important contributions by FXGO, RGW, JR, CFP, LB, KK, ST and TB. KK, ST, LB and TB designed the parent study. FXGO, RGW and JR were involved in collection, storage, laboratory testing and analysis of data from the parent study. MR, FXGO, RGW, JR, CFP, LB, KK, ST, TB and LCK contributed to the interpretation of the findings, critical review of the manuscript and approval of the final manuscript as submitted.
